# Comparison of Word Embeddings for Extraction from Medical Records

**DOI:** 10.3390/ijerph16224360

**Published:** 2019-11-08

**Authors:** Aleksei Dudchenko, Georgy Kopanitsa

**Affiliations:** 1 National Center for Cognitive Technologies, ITMO University, 197101 Saint-Petersburg, Russia; georgy.kopanitsa@gmail.com; 2 Institute of Medical Biometry and Informatics, Heidelberg University, 69120 Heidelberg, Germany

**Keywords:** word embedding, data extraction, machine learning, medical records

## Abstract

This paper is an extension of the work originally presented in the 16th International Conference on Wearable, Micro and Nano Technologies for Personalized Health. Despite using electronic medical records, free narrative text is still widely used for medical records. To make data from texts available for decision support systems, supervised machine learning algorithms might be successfully applied. In this work, we developed and compared a prototype of a medical data extraction system based on different artificial neural network architectures to process free medical texts in the Russian language. Three classifiers were applied to extract entities from snippets of text. Multi-layer perceptron (MLP) and convolutional neural network (CNN) classifiers showed similar results to all three embedding models. MLP exceeded convolutional network on pipelines that used the embedding model trained on medical records with preliminary lemmatization. Nevertheless, the highest F-score was achieved by CNN. CNN slightly exceeded MLP when the biggest word2vec model was applied (F-score 0.9763).

## 1. Introduction

This paper is an extension of the work originally presented in the 16th International Conference on Wearable, Micro and Nano Technologies for Personalized Health [1]. According to Dhamdhere et al., 80% of all healthcare data is unstructured [2]. Besides MRI, CT-scan, and imaging, a large amount of medical data collected within medical records is written in natural language. Moreover, despite using EMR, free text is still widely used because of some of its advantages such as more precise and flexible descriptions. However, free narrative text does not satisfy the criteria of semantic interoperability, cannot be analyzed by statistical tools, cannot be processed by a decision support system, and is not available for improving case-based systems. At the same time, the demand for medical free-text processing systems is increasing nowadays. 

There are two approaches to natural language text processing: a traditional one, based on language rules and domain ontologies, and methods that utilize machine learning. Machine learning algorithms show very good results in natural language processing (NLP) tasks [3,4,5,6], including data extractions [7] and named entity recognition [8,9,10]. 

A multi-layer perceptron (MLP) is an artificial neural network composed of an input layer to receive data, an output layer that makes a decision or prediction about the input, and hidden layers. The specific feature of this type of network is that each neuron from one layer is connected to every neuron from the next one. These layers are also called fully-connected or dense. As for any supervised learning technique, training involves adjusting the parameters, particularly the weights and biases, of the model in order to minimize error.

One of the successful approaches is to engage convolutional neural networks (CNN) for text classification [11]. CNNs have shown superior results in image recognition [12], and this neural network architecture can be applied to vector-represented text.

Convolutional neural networks (CNNs) [12,13] differ from fully-connected networks and have a more complex structure. CNNs have achieved state-of-the-art performance in image classification, speech recognition, and sentence classification. Convolution layers of CNNs, in contrast to fully-connected layers, are only connected to a small region of the previous layer and have a much smaller number of parameters. Technically, this allows us to use deeper models requiring the same memory but having better performance.

There are three main types of layers required to construct a CNN: the convolutional layer, pooling layer, and fully-connected layer. Convolutional layer parameters are a set of learnable filters. Each filter slides across the input data represented in the form of a matrix and computes a convolutional function. As a result, we obtain an activation map that gives the responses of a filter at every spatial position. In the process of training, the filters change to activate when they receive an input, which can be a feature of desirable output. Pooling layers are put in between convolutional layers to reduce the spatial size of the representation, reduce computation, and prevent overfitting. Pooling combines the outputs of neuron clusters into a single neuron as the maximum value of a cluster. Dense layers applied in CNN are the same layers as described in the MLP section. It makes the final prediction and returns the predicted class.

Long short-term memory networks (LSTMs) [14] are a type of recurrent neural network (RNN) that were developed to enable networks to take into account the information that has previously been fed into the network and can be useful for current predictions. RNNs are applied for sequential data such as time series, text, or any other sequence. In contrast to CNNs and conventional feed-forward networks, RNNs are able to model sequential data because they have a state variable to keep patterns in data. The state variables are updated over time. RNNs are able to predict the next value of the sequence. 

Advanced RNN LSTMs are widely used in language modelling and machine translation. LSTMs have better performance, providing more data and allowing for the stororage of memory for much longer than ordinary RNNs, and can also learn long-term dependencies. LSTM unit comprises a memory block—a cell and three regulators called gates: an input gate, an output gate, and a forget gate. The cells are responsible for remembering, and the gates manipulate this memory. The gates are continuous functions between 0 and 1 and the value controls how much information flows through the gate. 

All the neural networks mentioned above require text represented in some digital form. Word representation is an important and non-trivial task in NLP [15]. Good representation should preserve semantics and word context in a language. Word2vec [16] is a technique used to learn word embedding or feature representation of words. Word2vec is a distributed representation—the semantics of words is captured by the activation pattern of the resulting vector in contrast to a more simple one-hot representation. Word2vec can analyze a given text corpus and obtain numerical representations—vectors—such that words occurring in similar contexts have similar numerical representation. For vectors, this means that two vectors will be located closer to each other in a multidimensional vector space for words from similar contexts. Here, semantic meanings of words are learned from the context and words from similar contexts having similar semantics; as a result, their vector representations are located close to each other. 

Another issue is that the algorithm requires large labelled datasets for learning like any supervised learning algorithm does. Collecting such datasets is a hard and complicated task, taking a lot of human time and effort. 

Existing works report about successful results in different NLP applications in medicine. Danilov et al. [17] obtained 2.8 days mean absolute error (MAE) in hospital stay prediction. Zhou et al. [18] achieved 93% accuracy for medical event detection in Chinese clinical notes. However, every reported system aims and is trained to solve its exact task. Additionally, despite the progress in NLP field, the question about better embedding is still open [19,20]. In this work, we implemented three different embedding models and compared their performance in application to neural network classifier. We developed a prototype of a medical data extraction system for extraction diagnosis from free medical records in Russian.

## 2. Materials and Methods 

### 2.1. Data 

We randomly selected 220 de-identified records from a public hospital in Saint Petersburg, Russia. All records were created between 2015 and 2017 and contain information about the patient, diagnosis, complaints, laboratory and instrumental test results, and treatments.

#### 2.1.1. Labelled Dataset Gathering

Diagnosis and complaint sections of the records were analyzed in order to identify the most frequent complaints and diagnoses. Twenty four entities were comprised into a list for further extraction. To simplify the collection of samples for the dataset, we implemented a system that worked in two modes: semi-automatic mode and manual mode. In the semi-automatic mode, the entities were searched as keywords and the user was asked to accept or reject the identified example. To avoid skipping some complicated entities, we implemented a mode for manual labelling. Here, the user can select the desired piece of text and mark it as a corresponding entity. In both modes, the marked snippet of text with a fixed size was put into the dataset. The dataset was a .csv file with text snippet-entity couples.

#### 2.1.2. Embedding Models

**Figure 1:**
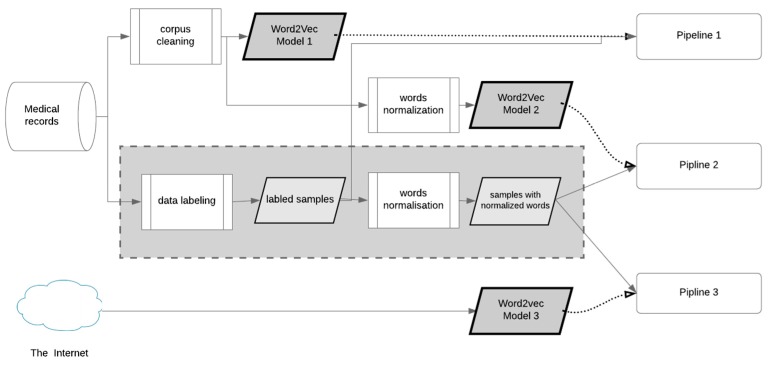
Word embeddings and samples’ preprocessing.

We used three different word embedding models. Two models were trained on our text corpora and one on a pre-trained model. This model was trained on the Russian National Corpus (RNC) [21] and texts from Wikipedia. This was the biggest model used in our research. In contrast, the other models were trained on much smaller corpora but related to the domain field. For this purpose, we gathered all medical records mentioned above and cleaned them by removing punctuation and filtering out non-alphabetic tokens. All tokens were converted to lowercase. Using this corpus, we trained the model shown in Figure 1 as word2vec model 1. For the other model, we normalized tokens—words in the corpus before training. For the normalization, we utilized pymorphy2, a morphological analyzer for Russian [22,23].

### 2.2. Preprocessing Pipelines

The general algorithm for extracting entities from narrative text in our work was as follows. We gathered snippets of text with labels and embedded each word from the snippet with the particular embedding model. Providing that we had three models, there were some different possibilities for word representation. Those possibilities were defined by the combination of snippet handling and applied embedding model. 

We implemented three data preprocessing pipelines. There were two types of sample preprocessing and three word2vec models. A combination of these two types of data was called a pipeline for the purposes of this study. In that context, a pipeline defined how samples from the dataset were represented to be fed to the classifier (Figure 1). Each pipeline had samples and embedding model as input. Dotted arrows were used for embedding data and solid arrows for samples. The grey rectangle on the figure highlights blocks responsible for samples preprocessing in contrast to blocks referring to word embedding. 

Pipeline 1 took samples from the dataset and represented each word according to embedding model 1. This embedding model was trained on the corpus of medical records without normalization. In the context of this work, normalization means retrieving a lemma of a word that is its dictionary or canonical form. This operation is also known as lemmatization. 

Pipeline 2, in contrast to the previous one, included normalization for embedding model and for samples in the dataset. Pipeline 3 took the same normalized samples as pipeline 2 but represented them as vectors using the pre-trained word2vec embedding model 3. 

Selection of these pipelines allowed us to answer the following questions: whether the pre-trained word2vec model gives better word representations for our task, and whether using word lemmatization gives better results. At the same time, these three pipelines covered all possible combinations of word embeddings and normalized/not normalized samples.

Data from every pipeline was fed to predictive models on the basis of three different types of neural networks: multi-layer perceptron, convolutional neural network, and long short-term memory networks. For model implementation, we used Python and Keras library (https://keras.io/), a high-level neural network API written in Python. Code can be found at https://github.com/AlekseiDudchenko/word2vec-models.

### 2.3. Algorithm Evaluation

To evaluate the prediction models, we applied the weighted average of precision and recall called F-score or F-measure (Equation (1)). The score was calculated globally by counting the total true positives, false negatives, and false positives. We implemented 10-fold cross-validation. The whole dataset was randomly split into 10 folds to train and evaluate the model 10 times on different sets of folds. The final score was calculated as the average F-scores of 10 obtained scores.

(1) $$Fscore=2\frac{precision*recall}{precision+recall}=2\frac{\frac{tp}{tp+fp}*\frac{tp}{tp+fn}\;}{\frac{tp}{tp+fp}+\frac{tp}{tp+fn}}.$$

## 3. Results

The list of entities for extraction comprised 24 items and the total of 983 samples. Those 24 entities were the more common diagnoses and complains in the processed records. Each sample in the obtained dataset was a pair of the snippet of text and the name of the entity. SNOMED codes of entities and their distribution in the dataset are shown in Table 1.

For word embedding, we applied three word2vec models. Table 2 provides the characteristics of the models. The “Corpus” column indicates what corpus was used to train the model, “Total words” indicates the total amount of words or tokens in the corpus, normalization is “yes” if tokens were normalized, “Words in the model” is the number of words included in the model and having vector representation, and “Vector size” is the dimensionality of the vectors in the model.

For model 1 and model 2, we used the same corpus of 220 medical records that was composed of 1,418,728 words in total. Model 3 was adopted without any changes. It was originally trained on a corpus of 788 million words. Words in the model were normalized. The final amount of vectors representing words in the model was 248,000.

According to the pipeline described in Section 2.2 of this work, model 1 corresponded to pipeline 1 and did not have normalization. Because of this, all words in the model remained in the form they had originally been in within the text. This led to a bigger amount of words in the resulting model. Model 1 had vectors for 7505 words, whereas model 2 was trained on the corpus of the same medical records but only had 3879 word representations. The corpus was normalized for model 2 before training, which decreased the amount of unique words.

Table 3 provides F-scores for three prediction models based on MLP, CNN, and LSTM, and the samples’ representation corresponded to three pipelines. The highest score (0.9763) was achieved with CNN and samples embedded with the biggest word2vec trained on RNC and Wikipedia (pipeline 3). 

**Figure 2:**
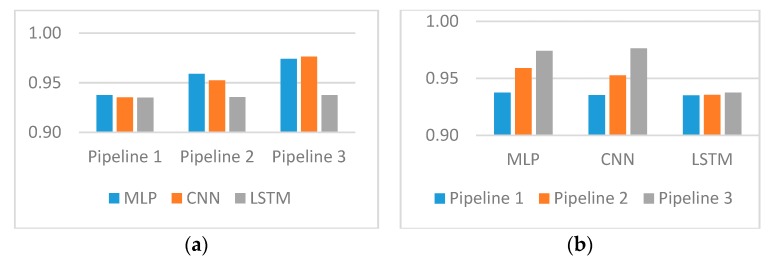
F-score. (**a**) grouped by pipelines; (**b**) grouped by predictive models

Scores grouped by pipelines and grouped by predictive models are shown in Figure 2. In this figure, it can be seen how better word representation influences the efficiency of each model (Figure 2a) and which model fits better for each pipeline (Figure 2b).

## 4. Discussion and Further Work

Lemmatization is often used in NLP, including preprocessing for the operation of word embedding. However, a lot of information goes missing while retrieving lemmas. We showed that lemmatization before obtaining a word2vec representation led to better performance for all three classifiers. When lemmatization was complete, the obtained model had much less elements but more contexts for more precise representation of each one.

One of the key points in our research was comparing different word2vec models in order to evaluate their efficiency in the task of entity extraction. The three pipelines used mostly differed in their word embedding models. Thus, we can make a conclusion regarding the models considering the results presented in Figure 2. The pre-trained model from the Russian National Corpus was the biggest model in our research and was applied within pipeline 3. The amount of words in the corpora dramatically exceeded the other two models. As a result, the score for the third pipeline achieved the highest values among all classifiers. Our word embedding models turned to be too small and the corpora that was collected for training word2vec models did not reach a sufficient scale. Due to this fact, we cannot make a conclusion about the better performance of embedding models trained on texts from the relevant field as compared with models trained on non-specific texts. To make such a conclusion, much bigger corpora of medical texts have to be used.

Three classifiers were applied to extract entities from snippets of text. MLP and CNN classifiers showed similar results to all three embedding models. MLP exceeded convolutional network on pipeline 2 that used the embedding model trained on medical records with preliminary lemmatization. Nevertheless, the highest F-score was achieved by CNN. CNN slightly exceeded MLP when the biggest word2vec model was applied.

LSTMs have a great potential in dealing with free text. However, the format of training data did not enable the advantages of these kinds of neural networks. Usage of LSTMs requires text data as sequences of tokens, not as snippets with fixed length.

The presented work is not yet a real-life task, but a simplification. This can be seen from the number of entities for extraction and the size of training dataset. The amount of entities was limited by 24, and all samples in the dataset were collected manually. We expect that results from real data would not be so high. To further improve this, the training dataset should be extended with more entities and more samples.

The next step in this direction is collecting a bigger comprehensive corpus of medical texts that includes not only records but also articles and guidelines. Having such a corpus, we will be able to train a word2vec model tailored for the medical field. The next step is the development of a complete system for data extraction with an increased size of the training dataset and user interface development. Dataset extension should be done in both directions by adding new entities and gathering more samples for each entity. Because only an entity having enough samples in the dataset can be successfully extracted, gathering a properly labeled dataset is the most challenging task. Nonetheless, such a system can be easily adopted and deployed within one particular department for structuring and second usage of medical data from unstructured narrative medical records.

## Figures and Tables

**Table 1:** SNOMED cods and samples distribution.

SNOMED Code	SNOMED Names	Count
443502000	Atherosclerosis of coronary artery (disorder)	235
57546000	Asthma with status asthmaticus (disorder)	11
72866009	Varicose veins of lower extremity (disorder)	22
4556007	Gastritis (disorder)	28
70153002	Hemorrhoids (disorder)	15
25064002	Headache (finding)	52
386705008	Lightheadedness (finding)	34
1201005	Benign essential hypertension (disorder)	125
84229001	Fatigue (finding)	71
235856003	Disorder of liver (disorder)	11
84089009	Hiatal hernia (disorder)	10
76581006	Cholecystitis (disorder)	18
45816000	Pyelonephritis (disorder)	29
44054006	Diabetes mellitus type 2 (disorder)	32
413838009	Chronic ischemic heart disease (disorder)	101
162864005	Body mass index 30+—obesity (finding)	25
266556005	Calculus of kidney and ureter (disorder)	20
298494008	Scoliosis of thoracic spine (disorder)	18
235494005	Chronic pancreatitis (disorder)	18
51868009	Duodenal ulcer disease (disorder)	24
191268006	Chronic anemia (disorder)	11
102572006	Edema of lower extremity (finding)	13
709044004	Chronic kidney disease (disorder)	34
(other)	Snippets without any disorder or finding	25

**Table 2:** Word2vec model parameters.

Embedding Model	Corpus	Total Words	Normalization	Words in the Model	Vector Size
Word2vec model 1	220 medical records	1,418,728	no	7505	50
Word2vec model 2	220 medical records	1,418,728	yes	3879	300
Word2vec model 3	Russian National Corpus (RNC) and Wikipedia	788,000,000	yes	248,000	300

**Table 3:** F-score.

Prediction Model	Pipeline 1	Pipeline 2	Pipeline 3
Multi-layer perceptron (MLP)	0.9374	0.9590	0.9741
Convolutional neural networks (CNN)	0.9353	0.9525	0.9763
Long short-term memory networks (LSTMs)	0.9351	0.9355	0.9375
